# Young children's learning of relational categories: multiple comparisons and their cognitive constraints

**DOI:** 10.3389/fpsyg.2015.00643

**Published:** 2015-05-19

**Authors:** Jean-Pierre Thibaut, Arnaud Witt

**Affiliations:** LEAD-Centre National de la Recherche Scientifique UMR-5022, Université de BourgogneDijon, France

**Keywords:** relational categories, relational language, comparisons, conceptual distance, conceptual development

## Abstract

Relational categories are notoriously difficult to learn because they are not defined by intrinsic stable properties. We studied the impact of comparisons on relational concept learning with a novel word learning task in 42-month-old children. Capitalizing on Gentner et al. (2011), two, three or four pairs of stimuli were introduced with a novel relational word. In a given trial, the set of pairs was composed of either close or far pairs (e.g., close pair: knife1-watermelon, knife2-orange, knife3-slice of bread and knife4-meat; far pair: ax-evergreen tree, saw-log, cutter-cardboard, and knife-slice of bread, for the “cutter for” relation). Close pairs (2 vs. 3 vs. 4 pairs) led to random generalizations whereas comparisons with far pairs gave the expected relational generalization. The 3 pair case gave the best results. It is argued that far pairs promote deeper comparisons than close pairs. As shown by a control experiment, this was the case only when far pairs display well known associations.

## Introduction

In the novel word learning literature, a broad distinction is made between object categories and relational categories. Object categories are often defined around shape commonalities which are known to be highly salient whereas other commonalities (e.g., texture, color, size) are less salient (Jones and Smith, 1993). Names for common object categories are acquired and produced very rapidly, right at the onset of word learning (Poulin-Dubois et al., 1999; Golinkoff and Hirsh-Pasek, 2008), before the production of names for relational categories.

Relational nouns refer to categories which are defined by relations between objects rather than by the intrinsic properties of the objects involved in these relations. For example, “neighbor” is not defined by any set of intrinsic properties which would characterize the entity it is applied to, but is defined by a relational structure referring to a particular type of relation, “something which is close,” between entities. Neighbor can refer to objects, persons or even to abstract entities such as events. In general, it has been shown that in tasks requiring detection of common relations such as analogical tasks, younger children often prefer object matches over relational matches (e.g., Gentner and Toupin, 1986; Richland et al., 2006; Thibaut et al., 2010). Specifically, relational categories appear later than many object categories because they are not defined by intrinsic, perceptually stable properties. Gentner et al. (2011) mention that using the MacArthur Communicative Developmental Inventory database reveals that entity nouns are frequent in the 8- to 16-month period whereas relational nouns appear in the 17–30-month range.

Moreover, previous studies showed that children might first misunderstand relational terms as referring to object categories (e.g., Hall and Waxman, 1993), because they focus on the object(s) properties at the expense of the relations connecting them. Thus, understanding which factors promote the relation-based abstraction and generalization is essential. In a recent paper, Gentner et al. (2011) suggested that the acquisition of relational terms might benefit from both syntactic support and comparisons between instances.

In their paper, Gentner et al. (2011) tested which conditions would lead to better generalization of relational nouns such as in “*X is the dax for Y*.” They introduced one (Experiment 1) or several (Experiments 2 and 3) learning pairs built around two familiar objects connected by a familiar relation (e.g.,*“cutter for”*), one being the operator (e.g., a knife), the other the entity (e.g., a watermelon). In all the experiments, the authors contrasted a Relational Label (e.g., *“the knife is the dax for the watermelon”*) and a No Label conditions (e.g., *“the knife goes with the watermelon”*). At test, in all the experiments, an entity (e.g., a sheet of paper) was introduced with three alternatives (i.e., a relational match– a pair of scissors–, a taxonomic match– a pile of sheets of paper–, and a thematic match– a pencil–). Children were asked to show which stimulus among the alternatives is the *dax* for the piece of paper (Relational Label condition) or which one goes with the piece of paper (the No Label condition). Overall, it was found that Relational Label conditions elicited more relational matches than the No Label conditions. However, the most effective condition was a progressive alignment relational label condition (Experiment 3), in which the authors introduced two pairs which were highly similar (called “*close pairs*” knife1-watermelon and knife2–orange) followed by the two new pairs which were less similar (called “*far pairs*” e.g., ax-evergreen tree and saw-log). In this case, even the 3-year-olds choose the relational alternative over the other alternatives beyond chance whereas in the other experiments only 4-year-olds or older children chose the relational choice. In brief, the most effective design was a progressive alignment condition combining multiple comparisons and two conceptual distances (close and far) between instances. The present study systematically manipulates the number of training items and the conceptual distance between them in order to understand the respective contribution of these factors and to find which conditions promote better learning performance through a generalization task.

Progressive alignment is a constructive way of obtaining generalization. The idea is to first implement a conceptual representation through a series of comparisons starting with easy commonalities, that is comparisons involving close pairs. The generalization scope of the category is then extended by progressively presenting dissimilar training exemplars. Another related use of progressive alignment is to provide intermediate steps (Ni) between a first training example (N1) and the generalization target (Nt), with Ni being closer to Nt than N1 is close to Nt. These intermediate steps allow participants to progressively connect far instances of a given concept.

Progressive alignment is a particular case of a broader approach which is based on comparison between category members. This comparison approach states that comparing stimuli generates better stimulus encoding and promotes generalization of non-salient dimensions or relations. Indeed, recent studies suggest that showing simultaneously several items belonging to the same category gives better understanding of taxonomical relations or of non-salient relevant dimensions than when the same stimuli are introduced in a no-comparison context (i.e., one after the other or one stimulus only) (Tversky, 1977; Thibaut, 1991; Gentner and Namy, 1999; Namy and Gentner, 2002; Graham et al., 2010; Augier and Thibaut, 2013). Comparisons would highlight common properties, especially non-salient properties, such as relational commonalities similar to the ones described above (*“is the dax for”*), that would be ignored if the stimuli were shown one by one (e.g., Gentner and Namy, 1999). To illustrate, Gentner and Namy (1999) focused on novel name learning (e.g., *dax*). One training stimulus (no-comparison case) or several training stimuli (comparison case) were introduced together with a novel name. In the test phase, children had to choose between a taxonomic match (i.e., an item belonging to the same taxonomic category as the training stimuli) and a perceptual match (i.e., an item perceptually similar to the training stimuli but coming from a different taxonomic category) the one they considered to be also a *dax*. Results show that children in the comparison case (e.g., a bicycle and a tricycle) preferentially chose the perceptually different taxonomic match (e.g., a skateboard) over the perceptual match (e.g., eyeglasses). By contrast, in the no-comparison condition in which only one training stimulus was introduced (e.g., one bicycle), the label *dax* was extended to the perceptual match (eyeglasses). Comparisons have been shown to favor generalization for different types of stimuli and situations in both adults and children. In a developmental context, demonstrations have been given for object names (e.g., Gentner and Namy, 1999; Namy and Gentner, 2002; Graham et al., 2010; Namy and Clepper, 2010), names for parts (Gentner et al., 2007), action verbs (Childers, 2011), adjectives (Waxman and Klibanoff, 2000), relational words (Gentner et al., 2011) or perceptual categories (e.g., Thibaut, 1991; Hammer et al., 2008, 2009; Andrews et al., 2011; Augier and Thibaut, 2013). For this reason, we decided to use the comparison paradigm described above in the context of a relational noun learning task, as in Gentner et al. (2011).

Before we come to the specifics of our study, we want to stress another dimension of comparison tasks. Although the no-comparison case provides limited information regarding category scope, it is the less cognitively demanding learning-generalization design since only one stimulus has to be processed. By contrast, if one defines cognitive complexity as the number of sources of variation to be related and processed in parallel (Zelazo and Frye, 1998; Andrews and Halford, 2002), comparison conditions are more cognitively demanding than no-comparison conditions. Indeed, to be effective, comparisons require systematic explorations of the stimuli in order to find common and/or distinctive features. However, some of the salient features that are noticed might be conceptually irrelevant in the trial context. Thus, it might be necessary to inhibit them in order to find common, relevant features. That is where the influence of cognitive flexibility comes in. If one first focuses on a dimension which cannot unify the entire set of stimuli included in a trial, one will have, later on, to focus on other dimensions, potentially unifying the stimuli. Thus, cognitive flexibility is involved in the sense that if participants spontaneously start to focus on an irrelevant dimension, they will eventually have to shift toward other dimensions in order to build a unified description of the stimuli (Anderson, 2002; Diamond, 2013). Following Augier and Thibaut (2013), we hypothesize that the cognitive costs associated with comparisons will increase as a function of the number of stimuli to be compared (Zelazo et al., 1997; Andrews and Halford, 2002; Diamond, 2013). Applying this line of reasoning in a comparison task, Augier and Thibaut (2013) manipulated the number of training stimuli (1 vs. 2 vs. 4) which were shown to 4- and 6-year old children. They showed that only 6-year-old children benefited from a larger number of stimuli (4 vs. 2 stimuli), whereas 4-year olds obtained the same performance in the 2 and 4 stimuli conditions. Given that executive functions develop very slowly and are less developed in young children (e.g., Anderson, 2002; Zelazo and Müller, 2010; Diamond, 2013), comparison costs are thus a very important issue to investigate in young children's conceptual learning.

The present study was derived from this executive-function comparison cost view. We started with Gentner et al. (2011) comparison condition (*close* vs. *far* cases) and systematically manipulated the number of pairs (2 vs. 3 vs. 4) which were introduced to illustrate the relational noun reference. We focused on 3-year-old children who, in Gentner et al. (2011), were beyond chance only in the progressive alignment condition (Experiment 3). The idea was to find other conditions that might promote relational generalization in comparison situations. First, we hypothesized that conceptual distance between the pairs (*close* vs. *far*) might interact with the number of training pairs. Unifying two close learning pairs is easy (e.g., knife1-watermelon and knife2-orange). Increasing the number of close pairs might be cognitively less detrimental because it is easier to find commonalties between pairs in the case of close pairs rather than in the case of far pairs. Indeed, it has been shown that analogies defined around pairs from the same conceptual domain are easier than analogies defined around pairs from distant conceptual domains (Gick and Holyoak, 1980; Green et al., 2010). By contrast, being confronted to a larger number of close pairs might contribute to underspecify the targeted relation. This would lead to narrower generalization in the sense that, a conceptually remote transfer example would be considered as “beyond the generalization scope” of the learning pairs. For example, showing four close pairs involving a knife and an object to be cut might favor a knife-interpretation of the “*is the dax for*” expression more than the corresponding two-close-pair case would do. For example, Namy et al. (2007) have shown that participants who viewed similar but different exemplars of a category during learning later classified objects based on conceptual commonalities. However, those who saw nearly identical exemplars classified objects based on perceptual similarity. In the lexical learning literature, it has been shown that children trained with variable exemplars generalized to novel exemplars of these categories and developed a discriminating word-learning bias more than children who were trained with less variable exemplars (Perry et al., 2010). Thus, there is evidence for better lexical generalization when variable, diverse, training exemplars are provided (see also Son et al., 2012, for discussion).

The *far* pair case is compatible with two opposite predictions. On the one hand, as in Augier and Thibaut (2013), adding more dissimilar pairs might lead to more converging evidence that will strengthen the interpretation of the conceptual relation. On the other hand, increasing the number of far pairs might lead to more information to integrate and cognitive overload and, thus, worsen performance. It might also be that there is an optimal number of pairs which might interact with conceptual distance (*close* vs. *far*). In short, increasing the number of close pairs might lead to poorer generalization of relational nouns, while increasing the number of far pairs might improve generalization performance beyond chance. A crucial issue is how the number of training items, thus the cognitive costs, will interact with this distance factor. Indeed, increasing the number of pairs might not lead to a linear increase of relational choices in the test phase.

## Materials and methods

### Participants

One hundred forty-four 42-month-old typically developing kindergarten children (71 female and 73 male) participated in this experiment (*M* = 3;6, range = 2;10–4;2). All were native French speaking children from the Dijon area (France). The children were randomly assigned to one of the six experimental conditions (*N* = 24 for each condition) which were compared; Distances (2: *close* vs. *far*) x Number of pairs (3: 2 vs. 3 vs. 4). Table 1 presents the characteristics of the groups. Children were tested individually in a quiet room of their school. This experiment was conducted in accordance with the ethical standards set out in the 1964 Declaration of Helsinki and written parental consent was obtained for each child.

**Table 1:** **Characteristics of the groups (F = female, M = male)**.

**Conceptual distance**	**Mean age (years, months)**	**Sex (F–M)**	**Number of pairs**	**Mean age (years, months)**	**Sex (F–M)**
	3;6 (range: 2;10–4;2)		Two	3;6 range: (2;10–4;2)	9–15
Close		34–38	Three	3;7 range: (2;11–4;2)	10–14
			Four	3;6 range: (2;10–4;2)	15–9
	3;6 (range: 2;10–4;2)		Two	3;6 range: (2;11–4;2)	12–12
Far		37–35	Three	3;6 range: (3;1–4;0)	12–12
			Four	3;6range: (2;11–4;2)	13–11

### Materials

**Figure 1:**
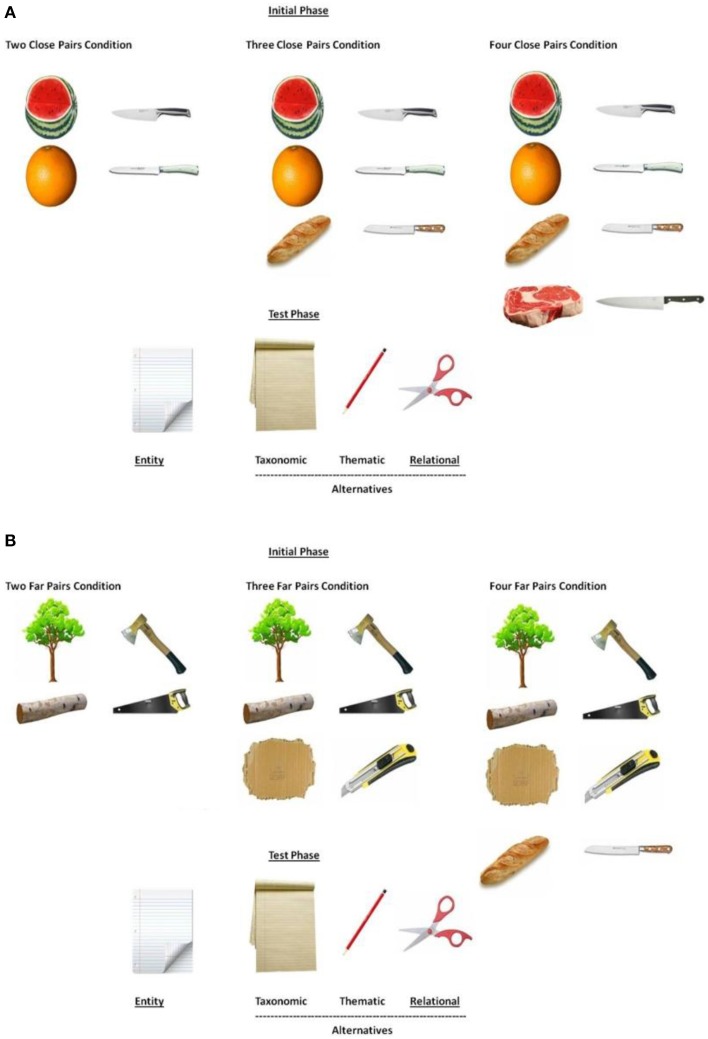
**Sample set depicting the *cutter for* relation, in the close (A) and far (B) conditions, inspired by Gentner et al. (2011)'s Experiment 3**.

Materials were adapted from Gentner et al. (2011)'s Experiment 3. Five sets of pictures were built. Each set corresponded to one of the five relational categories, *cutter for, home for, food for baby of*, and *container for*. Each set was composed of 20 cards, 16 training cards and 4 test cards (100 cards in total). Each card displayed one entity. The 16 training cards were composed of 4 *close* training pairs, and 4 *far* training pairs. As mentioned above we manipulated the number of training pairs (2, 3 or 4). Thus, a trial was composed of 2, 3 or 4 pairs (either *close* or *far*) depending on the condition (2-, 3-, 4-pair condition, respectively). Each pair was composed of an operator associated with an entity, (e.g., a knife as an operator and an orange as an entity). For each relational category (e.g., cutter for), the *close* pairs were composed of conceptually similar items (e.g., knife1-watermelon, knife2–orange, knife3-slice of bread, and knife4-meat), while the *far* pairs were composed of less conceptually similar pairs (e.g., ax-evergreen tree, saw-log, cutter-cardboard and knife-slice of bread). The 4 test cards consisted of an entity card choice (e.g., sheet of paper), a taxonomic card choice (e.g., pile of sheets of paper), a thematic card choice (e.g., pencil), and a relational card choice (e.g., scissors). Figure 1 depicts the *close* and *far* pairs used to instantiate the ″*cutter for*″ relation during the initial phase, as a function of the number of pairs presented to the participants, and the 4 response cards introduced at test (see Supplementary Material for the complete list of materials).

Instructions were identical to those in Gentner et al. (2011). We forged 5 different bisyllabic labels (pseudo-words) which are, as shown by Gathercole and Baddeley (1993), easier to remember than monosyllabic pseudo-words: buxi, dajo, zatu, xanto, and vira. Syllables were of the CV type which is the dominant word structure in French (from the “French database, Lexique.org,” New et al., 2004). The pictures used in our experiment also differed from that employed in Gentner et al. (2011). We used photos rather than line-drawings. However, we selected realistic pictures similar to those used in the original studies^1^. Independent similarity ratings from 54 students confirmed that *close* training pairs were more similar one to the others than *far* training pairs [see Supplementary Material for instructions; similarity ratings: *close* pairs (*M* = 5.94, *SD* = 0.40) vs. *far* pairs (*M* = 4.90, *SD* = 0.75), *t*_(29)_ = 5.93, *p* < 0.00001, *d* = 1.73]. We also compare the similarity between entities (e.g., similarity between orange, watermelon, slice of bread and meat (close case), or between ever-green tree, log, cardboard and slice of bread (far case) and between operators in the close and the far pairs [e.g., similarity between knife1, knife2, knife 3 and knife4 (close case), or between ax, saw, cutter and knife (far case), see Supplementary Material for details]. *Close* entities (*M* = 5.36, *SD* = 0.45) were significantly more similar than *far* entities (*M* = 2.76, *SD* = 0.51), *t*_(29)_ = 8.64, *p* < 0.00001, *d* = 5.41, and *close* operators (*M* = 5.37, *SD* = 0.49) were significantly more similar than *far* operators (*M* = 3.48, *SD* = 0.58), *t*_(29)_ = 5.92, *p* < 0.00001, *d* = 3.52).

### Procedure

Our procedure was as close as possible to that used in Gentner et al. (2011)'s Experiment 3. We illustrate it with the “*cutter for*” relational category. During the initial and test phases, the experimenter kept the speech flow constant, across items and experimental conditions (i.e., participants). Prosodic emphasis was added for the pseudo-words so that children noticed that the same label was used for each instance pair of stimuli.

#### Initial phase

A puppet named Sammy was used in order to make the task more attractive for children. The experimenter introduced the game with the following instructions (the example is for the four-close-pair condition; the instructions were the same for the far-pair conditions) “Hello, we are going to play a game together. In this game we are going to teach Sammy the word *buxy*. We are going to show him what *buxy* means.” “Look! This knife (the knife1 was put on the table) is the *buxy* for the watermelon (the watermelon was put on the table, left side of the knife1).” “This knife (the knife2 was put on the table, below the knife 1) is the *buxy* for the orange (the orange was put on the table, left side of the knife 2).” “This knife (the knife 3 was put on the table, below the knife 2) is the *buxy* for bread (the bread was put on the table, left side of the knife 3).” “This knife (the knife4 was put on the table, below the knife 3) is the *buxy* for the meat (the meat was put on the table, left side of the knife 4).” In the two-pair condition (either *close* or *far*) the training phase stopped after the second pair, after the third pair in the three-pair condition (either *close* or *far*) and after the fourth pair in the four-pair condition. The first training pair(s) remained in view until all the training pairs defining one trial had been showed.

#### Test phase

The test started with these instructions: “Now let's look all of them (gesturing across all the training pairs). You see how these (gesturing across all (two, three or four) operators) are *buxies* for these (gesturing across all (two, three or four) entities)? Now it's your turn. Which one of these (pointing to the test cards –taxonomic: pieces of paper; thematic: pencil; relational: scissors– were put on the table side by side below the training cards) is the *buxy* for the paper (the entity card –paper– was put on the table) in the same way?”

Children chose among the three test cards by pointing which is the *buxy* for the paper. This procedure was repeated for the 5 experimental relational categories. The order of presentation of the relational categories and the position of the three choices (left, middle or right) were counterbalanced, and the labels were interchanged among pairs across participants.

Importantly, even though the present experiment was adapted from Gentner et al. (2011)'s Experiment 3, we did not use the progressive alignment design used by the authors which consisted in, first, introducing close training pairs then far training pairs, in order to extend the scope of the category. In our experiment, close and far pairs were never mixed within the same training phase.

### Coding and analysis of the data

The extent to which children learned relational categories during the initial phase was assessed by coding the proportion of relational choices made at test, as well as the proportions of alternative choices (taxonomic and thematic choices). For each participant, the number of relational choices was calculated and the proportion of relational choices was computed on the basis of the five items presented during the task. A 2 × 3 ANOVA with Distance (2: *close* vs. *far*) and Number of pairs (3: 2 vs. 3 vs. 4) as between-subjects factors was carried out on the proportions of relational choices. Furthermore, we compared the proportions of relational responses to chance level performance (33%), using Student's *t*-tests. We also tested to what extent increasing the number of pairs (*close* or *far*) would lead or not to a linear increase of relational choices.

## Results

A 2 (Distance: close or far) × 3 (Number of pairs: 2 or 3 or 4 pairs) between-group analysis of variance^2^ (ANOVA) was carried out on the proportions of relational choices. It revealed a significant main effect of Distance with more relational choices for far pairs (*M* = 0.48; *SD* = 0.22) than for close pairs (*M* = 0.35; *SD* = 0.22), *F*_(1, 138)_ = 12.65, *p* < 0.0006, η^2^ = 0.08. The effect of Number of pairs was marginally significant, *F*_(2, 138)_ = 2.38, *p* = 0.095, η^2^ = 0.03, (two pairs, *M* = 0.40; *SD* = 0.26; three, *M* = 0.47; *SD* = 0.21; four, *M* = 0.37; *SD* = 0.20), and the Distance^*^Number of pairs interaction effect was not significant, *F*_(2, 138)_ = 0.006.

**Figure 2:**
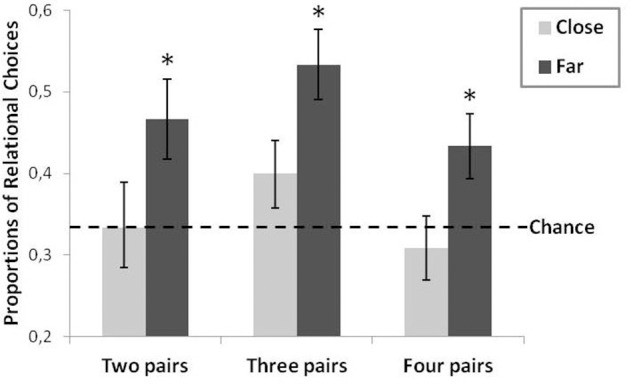
**Proportions of relational responses as a function of Type (2: Close vs. Far) and Number of pairs (3: two vs. three vs. four pairs)**. The error bars correspond to one standard error, the asterisks indicate a statistically significant comparison with chance, and the hatched line represents chance level (0.33).

Results are presented in Figure 2.

Student's *t*-tests were run to compare observed proportions of relational choices with the proportion corresponding to chance level performance (33%), for each of the six conditions (2, 3, 4 Close pairs and 2, 3, 4 Far pairs). In the *close* pair conditions, relational choices were not significantly above chance, whatever the number of pairs introduced during the initial phase: two (*M* = 0.33; *SD* = 0.27), *t*_(23)_ = 0, three (*M* = 0.40; *SD* = 0.20), *t*_(23)_ = 1.67, *p* = 0.11, or four pairs (*M* = 0.31; *SD* = 0.20), *t*_(23)_ = −0.63. By contrast, relational choices were significantly above chance in the three *far* pair conditions: two (*M* = 0.47; *SD* = 0.24), *t*_(23)_ = 2.71, *p* < 0.02, *d* = 0.78, three (*M* = 0.53; *SD* = 0.21), *t*_(23)_ = 4.67, *p* < 0.0002, *d* = 1.35, or four pairs (*M* = 0.43; *SD* = 0.19), *t*_(23)_ = 2.19, *p* < 0.02, *d* = 0.73 These results showed that generalization took place only in the far-pair conditions, which were above chance level.

These results confirmed those obtained with comparisons against chance. Thus, conceptual diversity at the encoding stage promotes relational abstraction. In addition, Figure 2 shows that the number of pairs also influenced generalization performance. We thus decided to test the trend of the curves depicted in Figure 2. A subsequent polynomial analysis (Howell, 2007) on the entire set of data (close and far pairs) revealed a non-significant linear effect, *F*_(1, 138)_ = 0.42, confirming that performance did not increase linearly with the number of pairs, whereas the quadratic trend was significant, *F*_(1, 138)_ = 4.36, *p* < 0.04, η^2^ = 0.03, suggesting that the optimal number of pairs was three pairs.

In sum, the results showed that a multiple-comparison learning design benefited from conceptual diversity between training exemplars (far pairs). They also showed that increasing the number of exemplars was beneficial even though there was an optimal number of training pairs (three) as shown by the quadratic function relating the number of training pairs (2, 3, and 4) and generalization performance.

Focusing on the benefits of conceptual diversity between the learning (far) pairs, we performed a follow-up Experiment 1B with uncommon far pairs. Indeed, there are cases in which the two items in a pair illustrate the relation in a less familiar way. For example, knife can be a “cutter for” a rope, or a saw can be a “cutter for” a cake. Even though these associations are less familiar, they describe situations that are possible in the world and truly understandable. The purpose of this follow-up experiment was to study whether a far-pair comparison design based with less familiar pairs would elicit relational choices or not. Two opposite predictions can be put forward. On the one hand, uncommon far pairs might promote a deeper analysis of the pairs. This will lead to the abstraction of the relevant relation, as in the far case in the previous experiment. On the other hand, it might be that finding less obvious relations will increase cognitive costs and will lead to poor performance because participants will be unable to find the relation for each pair and build an abstract representation unifying all the pairs. As in the main experiment, we took 2, 3, or 4 pairs to illustrate the target relations. As far as we can tell, there is no reason to believe that these results would not be obtained with real objects (rather than pictures).

### Experiment 1B

We introduced a new version of “far” pairs in which the relation was illustrated by a pair in which the association between the entity and the operator (e.g., candies and a rabbit) is uncommon in the world, in the context of the target relation (e.g., “food for”). The question was whether these uncommon pairs would elicit relational choices. Even though, they were uncommon, children could understand them because the entity and the operator were quite common. Two opposite predictions can be made. Uncommon pairs might give poor performance because they are less well conceptually entrenched in long term memory and, for this reason, understanding the relation unifying the pairs will require a larger number of comparisons between pairs which will lead to worse performance. This prediction was motivated by Thibaut et al. (2010) showed that analogies constructed around pairs composed of weakly semantically associated items (e.g., “man-plate; pig-bucket”) were more difficult than analogies built around “strong pairs.” They argued that when the target relation was embedded in a less familiar pair, the items connecting them was more difficult to find because they were less obvious and, thus, required more search. Also, the items which composed the pair activated strongly associated terms which were, nonetheless, irrelevant in the context of the target concept that one would have to inhibit. The same reasoning can be followed for the uncommon pairs. By contrast, in general, comparison situations are known to promote deeper encoding (Gentner and Namy, 1999; Namy and Gentner, 2002). It might be that less familiar relations would promote deeper comparisons and, as a result, would lead to the encoding of the target relation. This experiment can also be seen as a generalization of Experiment 1A in the sense that if far pairs led to better performance than close pairs, it might be that this would also be the case for pairs which were less common far pairs (see Son et al., 2012). In sum, we believe that there are two forces that work in opposite directions, deep comparison-based encoding on the one end, and increasing-cognitive-cost comparisons of uncommon pairs on the other end.

**Figure 3:**
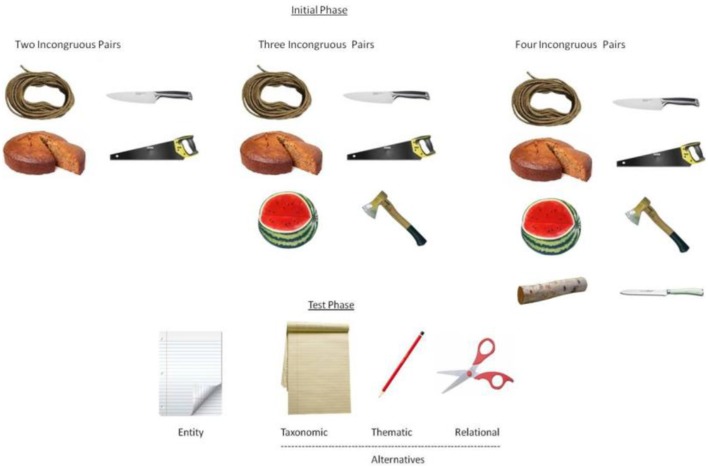
**Sample set depicting the *cutter for* in the ″less familiar″ pair condition**.

We manipulated the number of less-familiar pairs in the learning phase as in the main experiment, i.e., 2 vs. 3 vs. 4. The pairs were constructed in the same way as in the above case (see Figure 3). We asked 20 students to rate the familiarity of the pairs. Students were asked to rate close and far pairs from the above experiment and the novel uncommon far pairs (see above and Supplementary Material for the list of stimuli and the instructions). The familiar close and far pairs (overall, *M* = 6.47, *SD* = 0.33) were rated as significantly more familiar than the less-familiar pairs (*M* = 1.11, *SD* = 1.30), *t*_(19)_ = 18.32, *p* < 0.00001, *d* = 5.65.

The training and test procedures for the novel words were the same as in the previous experiment. Student's *t*-tests showed that children never produce relational choices beyond chance, whatever the number of training pairs: two (*M* = 0.34; *SD* = 0.19), *t*_(34)_ = 0.25, three (*M* = 0.29; *SD* = 0.23) *t*_(34)_ = −0.82, or four pairs (*M* = 0.37; *SD* = 0.18), *t*_(34)_ = 0.77.

We conclude that there is an optimal training diversity (far pairs rather than close pairs) that leads to an effective learning of relational categories. However, the present results show that uncommon pairs led to no learning probably because these pairs generate too many cognitive constraints in the task for the children, the nature of which will be discussed in the general discussion.

## General discussion

The present study sought to establish which conditions would promote relational concept learning in 42-month-old children and manipulated the number of exemplars available during training and the conceptual relatedness between the training pairs. We used a multiple comparison design which has been extensively used to study conceptual learning and which is known to promote better generalization performance (see Introduction, e.g., Gentner and Namy, 1999; Waxman and Klibanoff, 2000; Hammer et al., 2009; Childers, 2011; Augier and Thibaut, 2013).

Using Gentner et al.'s (2011) relational noun learning paradigm, manipulating the conceptual distance between the training pairs (*close* vs. *far*) and the number of compared items (2 vs. 3 vs. 4), we found that participants could abstract relational concepts, but only in a subset of experimental conditions which can be summarized in the following way. First, close pairs did not lead to the abstraction of the target relation: children did not select the relational choices over the other alternatives beyond chance, whatever the number of pairs. Second, by contrast, far pair conditions led to relational choices. Third, increasing the number of far pairs did not produce a linear increase of relational choices. Fourth, uncommon far pairs did not lead to any relational learning, whatever the number of training pairs.

### Conceptual relatedness: close and far training pairs

The first main factor that was hypothesized to influence generalization was the conceptual distance between the training pairs. Quite clearly, the close-pair training trials led to no relational abstraction. One interpretation of this failure is that the relevant relation that was abstracted during training was associated with characteristics of the training stimuli that were very specific to these stimuli. In the “knife” example, participants might have abstracted the relation “*cutter for involving a knife*” or “*a knife cutting something which is edible*.” Most likely, increasing the number of close instances did not improve performance because these instances led to an interpretation associated with the properties of the specific operators and entities which were used in the training examples. At test, the information associated with the relational choice (e.g., the scissors and sheet of paper) had nothing in common with the way the “*cutter for*” relation was instantiated in the training examples. At worse, it might well be that children used a pre-existing association between knife and the action of cutting something and did not really understand the word “buxy,” because the cutting event was very clear and familiar for them. In the “*far* case,” the conceptual distance between the training pairs in terms of the target relation did not lead to the construction of a specific association between the instances of the operator and/or the instances of the entity. In the case of the “*cutter for*” relation, introducing a knife and a watermelon together with an ax and an evergreen tree is not compatible with a specific cutter or any specific sharp entity and one has to find a decontextualized common relation. It is possible that, in the close case, no relation at all was abstracted, only common features such as “presence of a knife.” This view is consistent with Son et al. (2012) proposal and results. These authors provided children with different types of instantiations of a given schema (e.g., sharing) which they called unrelated, specifically-related and vaguely-related. They showed that a too specific schema, elicited no or poor transfer of the trained relation to perceptually-different exemplars, whereas they succeeded in the vaguely-related case (e.g., one puppet was surrounded by two abstract shapes). The authors argue that there is an optimal vagueness in training examples (somewhere in between too specific learning instances and too vague learning instances) that might foster relational learning. Along the same lines, Hammer et al. (2008) contrasted the contribution of the within- and between- class variations on the categorization of multidimensional stimuli. Within-category commonalities, which are relevant in the present context, (e.g., all A group members have property *i* and differ on their other properties), are clearly relevant in category learning. As mentioned by Hammer et al. (2008), when the distance between the compared exemplars increases in a multidimensional feature space, the comparison process is more informative for exemplars of the same class. This is compatible with the difference between the close and far conditions. However, as shown by Experiment 1B, one also has to consider the type of stimuli introduced in the learning phase. Moreover, these approaches do not account for the non-linear trend obtained here. These issues are now discussed.

### Comparison costs and the number of training exemplars

What the results show is that between the two-training-pair case, the less cognitively demanding learning-generalization design, and the four-training-pair case condition, the most cognitively demanding design in terms of the number of comparisons to be performed, the three-pair case led to a slightly better generalization performance. Why? As mentioned above increasing the number of standards improves the number of relevant information but also the number of sources of information to compare and integrate, thus the tasks cognitive demands (Zelazo and Frye, 1998; Andrews and Halford, 2002; Diamond, 2013). What the results suggest is that the three-pair condition was the best compromise between informativeness and cognitive demands. In the four-far-pair condition, participants have more pairs to decode, to keep in mind, and more pairs to compare and to integrate in a coherent representation, illustrating ″the best is the enemy of the good″ proverb. However, all the *far* conditions were beyond chance (see Fyfe et al., 2014, for a discussion of the role of abstraction and exemplars).

We believe that there is a trade-off between processing (comparing) the pairs and short-term storage of the information. Participants have to analyze the pairs in order to understand the relation but also to store this information in order to map it with the representation resulting from their analysis of the other pairs. Increasing the number of pairs means more pairs to process which, beyond a number of pairs, means less cognitive resources available to compare the pairs, or the other way round: more pairs to compare decreases the cognitive resources available to explore each pair. Children might also be less able to systematically perform within and between pair comparisons. Three pairs appeared to be the optimal number of training exemplars. However, there is no magic associated with three pairs. Our central hypothesis was that multiple comparisons would generate cognitive costs that would be handled more or less efficiently by executive functions, such as working memory, inhibition, and cognitive flexibility (e.g., Zelazo et al., 1997). Our results show that, with our design and stimuli, three turned out to be the optimal number of training items. In other situations, more training items might be optimum. Our point is not that there is a *magical* number of training items for all the situations but rather that, in many cases, providing more information through novel training exemplars will not lead to better results.

Our results are similar to what Augier and Thibaut (2013) found in their generalization task. The interaction between age (4- vs. 6-year olds) and number of stimuli showed that only 6-year-old children benefited from the most informative condition (4 vs. 2 stimuli). Importantly, the younger group obtained the same performance in the 2 and 4 stimuli conditions.

However, this discussion leaves open the question of the optimum diversity among the far pairs. One partial answer is given by our follow-up study which showed no preference for the relational solution in the case of less familiar training pairs. This suggests that processing uncommon instances of the same relation reduces the possibility to find the relation unifying each pair. This is analogous to what Thibaut et al. (2010) obtained with weak association analogies. Analogies constructed around less semantically associated pairs (e.g., “man-plate; pig-bucket” by contrast with the analogy “cow-milk; hen-egg” built around strongly associated pairs) were hypothesized to require more processing in order to find the relation between the items in the pair. As argued by Thibaut et al. (2010), when the target relation is embedded in a less familiar pair, the items in the pair will activate strongly associated terms which are, nonetheless, irrelevant in the context of the target concept. For example, in the case of “saw is the cutter for cake,” the item “saw,” for instance, will activate conceptually associated items such as “man” or “tree.” Since children have to find the relation between “saw” and “cake” and not between “saw” and “man” or “tree,” the latter items should be inhibited by the child in order to find a conceptual relation between “saw” and “cake.” Finally, it will also be more difficult to align the resulting representation of these uncommon pairs in order to find the common relation.

## Concluding remarks

In Gentner et al. (2011), the best generalization results were obtained with the progressive alignment design involving the presence of four standards (2 close first, then 2 far pairs), a result we replicated (see Footnote 1). This result does not run against our observation that 3 pairs rather than 4 was the optimal number in the far pair condition. First, progressive alignment is a general training format which differs from ours. Thus, the number of training of pairs cannot be directly compared. Moreover, the authors did not compare different progressive alignment conditions (e.g., providing 3 vs. 4 vs. 5 pairs), a comparison which was a central issue in our paper (see also Haryu et al., 2011, for a comparison of different progressive alignment conditions).

Future research will explore which type of information is important in the pairs and the role of conceptual distance between the pairs. One way to better understand how to build efficient learning comparison situations is to investigate which information is provided by similar and dissimilar standards. For instance, does progressive alignment rely on the use of different and complementary components of the generalization process through successive comparisons between a subset of highly similar standards that are followed by a subset of less similar standards? Or does progressive alignment work best when one gradually and smoothly reduces the distance between the standards and the target. Further studies systematically manipulating the distance between the standards and between the standards and the target stimuli to which the concept has to be generalized are necessary to address these issues.

### Conflict of interest statement

The authors declare that the research was conducted in the absence of any commercial or financial relationships that could be construed as a potential conflict of interest.

## Footnotes

^1^Note that we replicated Gentner et al. (2011)'s Experiment 3 with our experimental materials. We used the progressive alignment Relational language design depicted in the introduction section and in Gentner et al. (2011, pp. 1181–1182). Results showed that 3-year-old children (N = 18; M = 3;8, range = 3;0–3;11) choose the relational alternative (56%), over the other alternatives (21% for both taxonomic and thematic choices), and beyond chance (33%), p < 0.001. These results are quite similar to those obtained by Gentner et al. (2011). This replication shows that our materials and procedure were equivalent to Gentner et al.'s.

^2^Preliminary data analysis including the Gender factor revealed a significant effect of Gender, *F*_(1, 132)_ = 4.1, *p* = 0.045, η^2^ = 0.03, with boys outperforming girls, and a significant Gender^*^Distance effect, *F*_(1, 132)_ = 4.76, *p* < 0.04, η^2^ = 0.03, revealing a superiority of the boys only in the close pairs condition, while Gender^*^Number and Gender^*^Number^*^Distance interaction failed to show significant effects, respectively *F*_(2, 132)_ = 0.88 and *F*_(2, 132)_ = 0.01. In addition, when items (the five relational categories: home for, cutter of, baby of, food for and container for) were analyzed separately, Gender never reached significance, and never interacted with other factors (Distance, Number, or both). Because our a priori hypothesis did not predict any gender effect or any interaction effects with Gender, Gender factor was not included in the present analysis.
