# Supplementary material for: Young children's learning of relational categories: multiple comparisons and their cognitive constraints
Source: Front Psychol. 2015 May 19;6:643. doi: 10.3389/fpsyg.2015.00643 (PMC4436577; doi:10.3389/fpsyg.2015.00643)
Supplement: Supplementary file 1 [file DataSheet1.DOCX]

Appendix 1

*Complete list of materials for the close and far conditions*

Alternatives

Entity Operator Taxonomic Thematic Relational

Initial Phase (*home for*)

Close Exemplar 1 Man Home1

Close Exemplar 2 Woman Home2

Close Exemplar 3 Amerindian Tepee

Close Exemplar 4 Man2 Apartment

Far Exemplar 1 Bird Nest

Far Exemplar 2 Fish Fish bowl

Far Exemplar 3 Bee Swarm

Far Exemplar 4 Man Apartment

Test Phase Dog Dog Bone Doghouse

Initial Phase (*cutter for*)

Close Exemplar 1 Watermelon Knife1

Close Exemplar 2 Orange Knife2

Close Exemplar 3 Slice of bread Knife3

Close Exemplar 4 Meat Knife4

Far Exemplar 1 Evergreen tree Ax

Far Exemplar 2 Log Saw

Far Exemplar 3 Cardboard Cutter

Far Exemplar 4 Bread Knife

Test Phase Paper Paper Pencil Scissors

Initial Phase (*food for*)

Close Exemplar 1 Rabbit Carrot

Close Exemplar 2 Mouse Cheese

Close Exemplar 3 Squirrel Hazelnut

Close Exemplar 4 Guinea pig lettuce

Far Exemplar 1 Robin Worm

Far Exemplar 2 Penguin Fish

Far Exemplar 3 Turtle lettuce

Far Exemplar 4 Monkey Banana

Test Phase Horse Horse Saddle Hay

Initial Phase (*baby of*)

Close Exemplar 1 Cat Kitten

Close Exemplar 2 Dog Puppy

Close Exemplar 3 Lion Lion cub

Close Exemplar 4 Bear Bear cub

Far Exemplar 1 Deer Fawn

Far Exemplar 2 Woman Baby

Far Exemplar 3 Hen Chick

Far Exemplar 4 Cat Kitten

Test Phase Duck Duck Pond Duckling

Initial Phase (*container for*)

Close Exemplar 1 Coin Piggy bank

Close Exemplar 2 Bill Wallet

Close Exemplar 3 Coin2 Purse

Close Exemplar 4 Bill & coin Cash register

Far Exemplar 1 Shirt Closet

Far Exemplar 2 Milk Refrigerator

Far Exemplar 3 Exercise Books Schoolbag

Far Exemplar 4 Eggs Egg box

Test Phase Crayon Crayon Paper Crayon box

Appendix 2

*Similarity ratings between the close pairs and between the far pairs*

Participants were fifty-four undergraduate students enrolled in a Psychology course. Three groups of Participants (each N = 18) completed a Pair-Rating task followed by an Item-Rating task. In the Pair-Rating Task, participants rated on a 7-point scale the similarity between close pairs (e.g., knife1-watermelon, knife2-orange, knife3-bread-, knife4-meat) and between far pairs (e.g., ax-evergreen tree, saw-log, cutter-cardboard, knife-bread). In the Item-Rating task, participants rated the similarity between entities and operators in the close pairs (e.g., watermelon, orange; knife1, knife2) and in the far pairs (evergreen tree, log; ax, saw). In each of the three groups, each participant saw two of the 6 close and two of the 6 far pairs, so that similarity pair- and item-ratings were completed for all the possible pairs in the close and far pairs. The pairs were presented in pre-randomized orders.

The results revealed that the close pairs (*M* = 5.94, *SD* = .40) were rated as more similar than the far pairs (*M* = 4.90, *SD* = 0.75), *t*(29) = 5.93, *p* < .00001, *d* = 1.73. In addition, entities and operators in the close pairs (*M*_Entity_ = 5.36, *SD* = .45; *M*_Operator_ = 5.37, *SD* = .49) were rated as more similar than entities and operators in the far pairs (*M*_Entity_ = 2.76, *SD* = .51; *M*_Operator_ = 3.48, *SD* = 0.58), respectively *t*(29) = 8.64, *p* < .00001, *d* = 5.41 and *t*(29) = 5.92, *p* < .00001, *d* = 3.52.

Appendix 3

*Complete list of materials for the "less-familiar" condition*

Alternatives

Entity Operator Taxonomic Thematic Relational

Initial Phase (*home for*)

Less-familiar 1 Man1 Igloo

Less-familiar 2 Man2 Tepee

Less-familiar 3 Bee Nest

Less-familiar 4 Bear Log cabin

Test Phase Dog Dog Bone Doghouse

Initial Phase (*cutter for*)

Less-familiar 1 Rope Knife1

Less-familiar 2 Cake Saw

Less-familiar 3 Watermelon Ax

Less-familiar 4 Log Knife2

Test Phase Paper Paper Pencil Scissors

Initial Phase (*food for*)

Less-familiar 1 Rabbit Bonbons

Less-familiar 2 Mouse Cookie

Less-familiar 3 Bird Chocolate

Less-familiar 4 Penguin Hamburger

Test Phase Horse Horse Saddle Hay

Initial Phase (*baby of*)

Less-familiar 1 Dog Different breed puppy

Less-familiar 2 Chat Different breed kitten

Less-familiar 3 Lion White tiger cub

Less-familiar 4 White bear Brown bear cub

Test Phase Duck Duck Pond Duckling

Initial Phase (*container for*)

Less-familiar 1 Marbles Purse

Less-familiar 2 Exercise books Closet

Less-familiar 3 Choose Refrigerator

Less-familiar 4 Tennis balls Egg box

Test Phase Crayon Crayon Paper Crayon box

Appendix 4

*Familiarity and Plausibility ratings for the "less-familiar" pairs.*

Participants were fifty-six undergraduate students enrolled in a Psychology course. They were divided into two groups. The first group of participants (N = 28) completed a Familiarity Pair-Rating task. They were asked to rate the familiarity of (four) *"less familiar"* pairs (e.g., knife1-rope, saw-cake, ax-watermelon, knife2-log), (two) close pairs (e.g., knife1-bread and knife2-meat), and (two) far pairs (e.g., ax-evergreen tree and cutter-cardboard) on a 7-point scale. The pairs were presented in pre-randomized orders. The results revealed that the *"less familiar"* pairs (*M* = 1.11, *SD* = 1.30) were rated as less familiar than the close-far pairs (*M* = 6.47, *SD* = .33), *t*(19) = 18.32, *p* < .00001, *d* = 5.65.

The second group of participants (N = 28) completed a Plausibility Pair-Rating task. They saw the same set of *"less familiar"* pairs together with non-plausible pairs (e.g., glue stick and a piece of cardboard for the *cutter for* relation). They were asked to estimate whether or not these (*"less-familiar" vs. "non-plausible" pairs*) pairs were possible instantiations of the given relation (e.g., *cutter for*), by selecting the answer "*yes, in the world the relation x might connect these two items*" or "*no, this association does not account of this relation at all*". The analysis of the proportions of positive responses in this second task showed that the *"less-familiar"* pairs (*M* = 0.76, *SD* = .24) were rated as significantly more plausible instanciations of the five relations tested in this experiment than the non-plausible pairs (*M* = 0.03, *SD* = .04), *t*(19) = 13.29, *p* < .00001, *d* = 4.24.
